# Supplementary material for: Multiple Salivary Proteins from Aedes aegypti Mosquito Bind to the Zika Virus Envelope Protein
Source: Viruses. 2022 Jan 24;14(2):221. doi: 10.3390/v14020221 (PMC8876891; doi:10.3390/v14020221)
Supplement: Supplementary file 1 [file viruses-14-00221-s001.zip › viruses-1527024-supplementary-new.pdf]

Supplementary Information

Table S1: *Aedes aegypti* salivary proteins selected for cloning and expression in HEK293E cells.

Vector base	Type of protein	Protein size (Daltons)
AAEL000793	antigen 5 AAEL000793-PA [*Aedes aegypti*]	31897.04
AAEL008766	proline rich salivary secreted peptide [*Aedes aegypti*]	21117.95
AAEL002704	salivary anti FXa serpin [*Aedes aegypti*]	50903.6
AAEL007420	Fxa-directed anticlotting serpin-like protein [*Aedes aegypti*]	50392.91
AAEL000533	putative C-type lectin [*Aedes aegypti*]	20676.06
AAEL006347	2105426A apyrase	65730.01
AAEL006485	AAEL006485-PA purine hydrolase [*Aedes aegypti*]	40935.67
AAEL007394	short salivary D7 protein [*Aedes aegypti*]	21567.35
AAEL003057	AAEL003057-PB [*Aedes aegypti*]	32656.47
AAEL003107	AAEL003107-PA [*Aedes aegypti*]	41607.56
AAEL003182	AAEL003182-PA [*Aedes aegypti*]	50039.05
AAEL003601	AAEL003601-PA [*Aedes aegypti*]	39193.4
AAEL000726	putative secreted protein angiopoietin [*Aedes aegypti*]	36719.89
AAEL006406	putative 16.9 kDa secreted protein D7 [*Aedes aegypti*]	19939.63
AAEL024303	putative 18.2 kDa secreted protein [*Aedes aegypti*]	21163.03
AAEL024303	short D7 protein [*Aedes aegypti*]	21248.11
AAEL022638	putative 14.5 kDa salivary protein [*Aedes aegypti*]	20050.05
AAEL010235	30 kDa salivary gland allergen variant 2 [*Aedes aegypti*]	29154.89
AAEL006424	37 kDa salivary gland allergen Aed	36895.1
AAEL006417	37 kDa salivary gland allergen Aed 2	38627.77
